# Multiple differences in pathogen-host cell interactions following a bacterial host shift

**DOI:** 10.1038/s41598-020-63714-0

**Published:** 2020-04-22

**Authors:** Andrea J. Dowling, Geoffrey E. Hill, Camille Bonneaud

**Affiliations:** 1 Biosciences, College of Life and Environmental Science, Penryn Campus, University of Exeter, Cornwall, TR10 9FE UK; 2 Department of Biological Sciences, Auburn University, Auburn, AL36849-5414 USA

**Keywords:** Ecology, Evolution

## Abstract

Novel disease emergence is often associated with changes in pathogen traits that enable pathogen colonisation, persistence and transmission in the novel host environment. While understanding the mechanisms underlying disease emergence is likely to have critical implications for preventing infectious outbreaks, such knowledge is often based on studies of viral pathogens, despite the fact that bacterial pathogens may exhibit very different life histories. Here, we investigate the ability of epizootic outbreak strains of the bacterial pathogen, *Mycoplasma gallisepticum*, which jumped from poultry into North American house finches (*Haemorhous mexicanus*), to interact with model avian cells. We found that house finch epizootic outbreak strains of *M. gallisepticum* displayed a greater ability to adhere to, invade, persist within and exit from cultured chicken embryonic fibroblasts, than the reference virulent (R_low) and attenuated (R_high) poultry strains. Furthermore, unlike the poultry strains, the house finch epizootic outbreak strain HF_1994 displayed a striking lack of cytotoxicity, even exerting a cytoprotective effect on avian cells. Our results suggest that, at epizootic outbreak in house finches, *M. gallisepticum* was particularly adept at using the intra-cellular environment, which may have facilitated colonisation, dissemination and immune evasion within the novel finch host. Whether this high-invasion phenotype is similarly displayed in interactions with house finch cells, and whether it contributed to the success of the host shift, remains to be determined.

## Introduction

Novel disease emergence can occur when a microbial pathogen of one host jumps into a different host species^1,2^. While contact with the novel host is necessary for a jump to occur in the first place, the success of pathogen emergence then relies on the pathogen’s ability to infect and transmit within the novel host species^3,4^. For this reason, pathogen emergence is likely to be associated with changes at key cellular virulence mechanisms that facilitate tissue colonization and disease progression, including the adhesion, invasion of, replication within and exit from host cells and tissues^5,6^. Our understanding of these processes are largely based on emerging viral diseases^7^. However, novel bacterial outbreaks are also prevalent and predicted to increase in frequency as a result of current socio-demographic and environmental changes, as well as the apparent rise of increasingly virulent and opportunistic bacterial strains^8^. Identifying the phenotypic and molecular changes that enable bacterial pathogens to infect novel host species is therefore important for advancing our understanding of how and why novel bacterial outbreaks occur, as well as for designing effective control measures.

Mycoplasmas (class *Mollicutes*) are the smallest and simplest self-replicating bacterial pathogens, and economically important emerging and re-emerging pathogens of humans, agricultural animals and wildlife^9–12^. Having evolved from a Gram-positive ancestor by reductive evolution, mycoplasmas display very small genomes of ~1 MB and no cell wall^13^. The resulting loss of many biochemical pathways have made them into obligatory parasites, highly dependent on their host for provision of the substrates required for existence and typically displaying strict host and tissue specificity^14^. Their rapid adaptation to novel host environments, however, is made possible by exceedingly high rates of substitutions (0.8–1.2 × 10^−5^ per site per year^15^) that rival the highest yet reported in bacteria (*Helicobacter pylori*: 1.4 × 10^−6^ per site per year^16^). While much attention has been placed on the role of antigenic variation at the bacterial cell surface in host adaptation through immune-evasion^17^, comparative studies of mycoplasma isolates of differing virulence indicate that other processes will also affect infection success^18^. For example, the gene *mslA* encodes a lipoprotein that is implicated in the acquisition of nucleotides from the environment^19^ and *M. gallisepticum* mutants for that gene display decreased colonization success and reduced virulence in the chicken host^20^. Similarly, *M. gallisepticum* lacking the functional metabolic factor dihydrolipoamide dehydrogenase (Lpd), which is involved in the glycolysis pathway and production of ATP, have lower infection success, possibly because of energy shortage during host colonization^21^. The ability to bind and invade host cells is also likely to be critical to bacterial dissemination and hence disease initiation and progression. For instance, the virulent poultry strain of *M. gallisepticum* (R_low), but not the attenuated derivative (R_high), has been shown to adhere and invade chicken embryonic fibroblasts (CEF), HeLa cells and chicken erythrocytes^22,23^. Understanding why and how pathogenic mycoplasmas are currently emerging will require identifying how such virulence-associated traits change during host shifts.

*Mycoplasma gallisepticum* is a serious bacterial pathogen of poultry that jumped into a wild North American songbird, the House finch (*Haemorhous mexicanus)* in 1994^24,25^. Although this bacterium causes chronic respiratory disease and infectious sinusitis in its poultry hosts^26^, the jump to house finches was associated with a change in symptomology, with high mortality rates in house finches resulting from severe conjunctivitis leading to a reduced ability to feed and escape predators^27^. Phylogenetic evidence has shown that this was a unique host-shift event resulting from a single progenitor strain of *M. gallisepticum* that ultimately established in the novel house finch host^28^. Whole-genome sequence comparison of poultry and house finch epizootic outbreak strains revealed that the jump to house finches was associated, among others, with extensive changes at variable surface lipoprotein (*vlhA*) genes, as well as gene losses, such as the loss of the host specificity of DNA (*hsd*) locus thought to be involved in tissue tropism^29,30^, and smaller coding changes, notably at genes involved in cytoadherence, such as *hlp3*, *plpA*, *mgc2*, *gapA* and *crmB-like*^31^. Interestingly, Hlp3 and PlpA are fibronectin-binding proteins that bind to the extra-cellular matrix and that have been identified as important determinants of virulence^18^. Similarly, the loss of GapA has been shown to be associated with a loss of cytoadherence^32,33^ and recent evidence suggests that GapA may be required for the bacteria to reach, adhere to and persist in host tissue^34^.

Here, we investigate the ability of a house finch epizootic outbreak strain of *M. gallisepticum* to colonise, persist within and cause cytotoxicity in model avian cells. To do so, we compared the avian cell interactions of the outbreak strain of *M. gallisepticum* isolated in house finches in 1994 (HF_1994), with those of the reference virulent R type poultry strain (R_low) and the high passage, attenuated derivative of this strain (R_high). Given that we have no knowledge of the progenitor poultry strain at the origin of the house finch clade of *M. gallisepticum*, we cannot use R_low (or R_high) as a representative ancestral strain. Rather, R_low and R_high were chosen as references in our experiments because they are well characterised, and many of their virulence related attributes, representing those that we wished to investigate in the house finch strain, have been comprehensively studied^18,22,34–39^. We used the HF_1994 strain to characterise the virulence phenotype at the point of outbreak because it represents the earliest strain collected following first observation of disease in the house finch, and because its genome has been sequenced (GenBank accession number CP003506)^31^. Furthermore, we tested the generality of our key finding in two additional house finch early epizootic strains collected in 1995 and in 1996, thereby verifying that HF_1994 is not an anomalous outlier but indeed a valid representative of house finch strains of *M. gallisepticum* at epizootic outbreak. (Note that because *M. gallisepticum* has evolved rapidly following the jump into house finches^40,41^, particularly in response to the spread of host resistance^42^, we cannot use later epizootic strains to characterise the virulence phenotype at outbreak). Fibroblasts are one of the main structural cells of conjunctival tissues and the site of *M. gallisepticum* infection. A cultured chicken embryonic fibroblast cell line (DF-1) was therefore chosen as a simplified model and non-phagocytic avian cell environment to investigate the typical *M. gallisepticum* cellular infection phenotypes of adhesion, invasion, cell exit and cytotoxicity, which are all known virulence mechanisms of virulent poultry strains such as R_low^43^. Given that *M. gallisepticum* can invade a variety of avian and non-avian cells, such as chicken erythrocytes (both *in vitro* and *in vivo*)^23^, erythroblasts^44^, and embryonic fibroblasts^22^, as well as human (HeLa)^22,44^ and murine embryonic stem cells^44^, its invasion mechanisms have a broad host range and our measures in DF-1 cells are therefore likely to be indicative of ones occurring in avian cells more largely.

## Results

### The outbreak strain is highly adhesive and invasive

To investigate the ability of the epizootic outbreak strain, HF_1994, and of the two early epizootic strains HF_1995 and HF_1996, to invade avian cells, DF-1 cell monolayers were infected either with HF_1994, HF_1995, HF_1996, R_high or R_low. Adhesion and invasion of these avian cells were then examined using two complementary techniques. First, we used the qualitative differential immunofluorescence (DIF) assay, which allows the differential visualization of intra- and extra-cellular bacteria in infected avian cells. Second, we used the quantitative gentamicin invasion assay, which is based on antibiotic (gentamicin) treatment of infected avian cells to kill all the extra-cellular bacteria and thereby permits the detection of viable intra-cellular bacteria only^45,46^.

**Figure 1:**
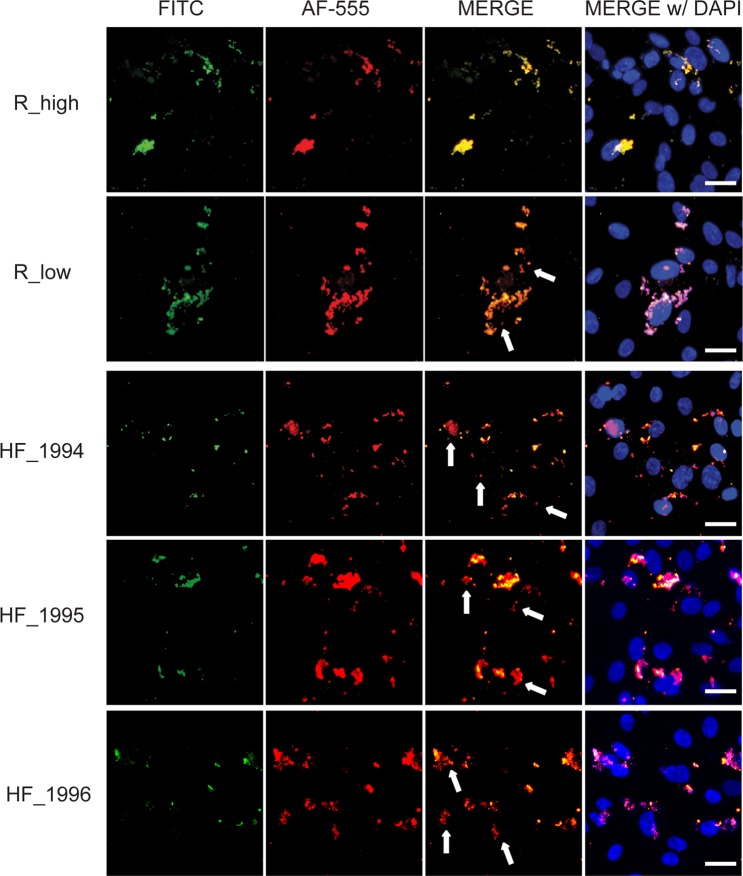
*M. gallisepticum* HF_1994 is highly adherent and invasive of non-phagocytic avian cells. Differential immunofluorescence staining images illustrating the interaction of *M. gallisepticum* strains from poultry (R_high and R_low) and from epizootic outbreak in house finches (HF_1994, HF_1995 and HF_1996) with DF-1 cells 16 h following infection. FITC panels show extracellular mycoplasmas labelled green, AF-555 panels show both intracellular and extracellular mycoplasmas labelled red, overlay panels are merged FITC and AF-555 images enabling identification of extracellular (yellow) and intracellular (red) mycoplasmas, further, merge w/DAPI includes nuclei labelled blue. Arrows indicate intracellular mycoplasma in R_low, HF_1994, HF_1995 and HF_1996 merge panels. Scale bars = 10 μm.

DF-1 cell monolayers were stained using the DIF technique 16 h post-infection. Cellular localization of the mycoplasmas in the infected DF-1 monolayers was analyzed by imaging the FITC-stained extracellular bacteria, and the Alexa Fluor 555 (AF-555) stained extra and intracellular bacteria through the respective filter sets (Fig. 1). The merge of FITC and AF-555 images revealed extracellular mycoplasmas (yellow) and intracellular mycoplasmas (red). This showed qualitatively: (i) adherence, but not invasion, of DF-1 cells by R_high; (ii) invasion of DF-1 cells by R_low; and (iii) greater invasion of DF-1 cells by HF_1994, HF_1995 and HF_1996 compared to R_low, as indicated by a greater extent of red mycoplasma-staining visible in the merged image (Fig. 1).

We quantitatively substantiated the high levels of invasion of outbreak strain HF_1994 and the early epizootic strains HF_1995 and HF_1996 respective to R_low using the gentamicin invasion assay. Invasion, cell association and adherence were measured 60 min post-inoculation of DF-1 cells, which is shorter than one generation time of the bacterium (estimated at >2 h). This ensures that our measures are not confounded by any difference in growth rate between the *M. gallisepticum* strains used.

**Figure 2:**
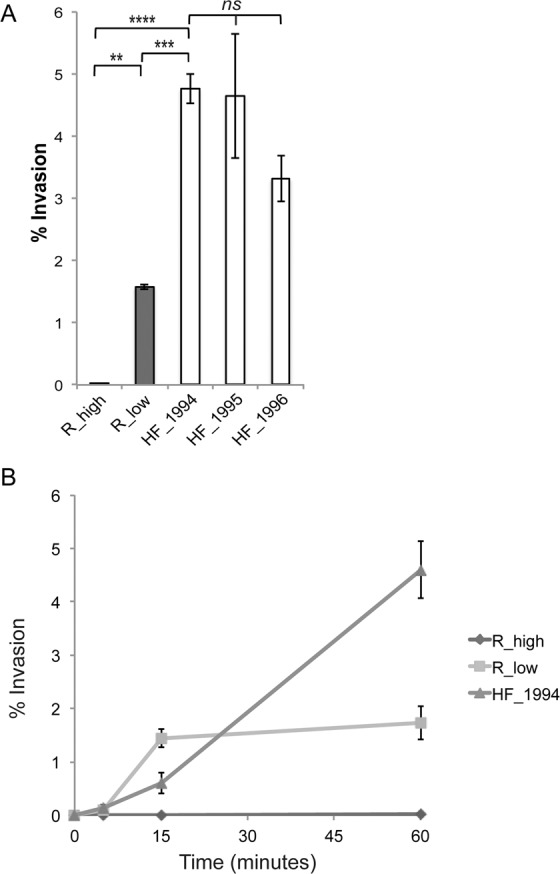
HF_1994 invades avian cells rapidly. (**A**) Percentage invasion of DF-1 cells relative to the initial inoculum obtained from gentamicin invasion assay of DF-1 cells infected with either R_high R_low, HF_1994, HF_1995 or HF_1996 for 1 h. * indicates p < 0.05, ** indicates p < 0.01, *** indicates p < 0.001, *** indicates p < 0.0001. (**B**) Time course of invasion of *M. gallisepticum* strains into DF-1 cells. DF-1 monolayers were inoculated with R_high, R_low or HF_1994 and incubated for set time points (5, 15 and 60 mins) prior to treatment with gentamicin. Percentage invasion was calculated relative to the initial inoculum.

Following treatment of DF-1 cell cultures with gentamicin, we determined the invasion frequency (percentage invasion) as the percentage ratio of the number of CFUs recovered post-treatment to the number of colonies present in the initial inoculum added to the DF-1 cells (Fig. 2A). There was a significant difference in the ability of the strains to invade DF-1 cells (one-way ANOVA: F_2,6_ = 102, p < 0.0001). Indeed, the 4.8% (±SE 0.5) of the initial HF_1994 inoculum that had invaded DF-1 cells was significantly higher than both the 1.8% (±SE 0.25) invasion frequency of R_low (Tukey HSD test: p < 0.001) and the 0.02% (±SE 0.03) invasion frequency of R_high (Tukey HSD test: p < 0.0001) (Fig. 2A). The two other early house finch epizootic strains, HF_1995 and HF_1996, displayed the same increased ability to invade avian cells as HF_1994 (Figs. 1 and 2A). Indeed, neither strain differed significantly in their invasion frequency relative from HF_1994 (Tukey HSD tests; HF_1995: p = 0.99; HF_1996: p = 0.29). In addition, the 4.7% (±SE 1) invasion frequency of HF_1995 and the 3.4% (±SE0.6) invasion frequency of HF_1996 were both significantly higher than that of R_low (Tukey HSD tests; HF_1995: p < 0.01; HF_1996: p < 0.01).

While the gentamicin invasion assay cannot alone be used to fully characterize quantitative differences in cell surface adherence between strains, we used it here to gain additional insights into the relative ability of HF_1994 to associate with our model avian cells. Following wash steps to remove all non-adherent mycoplasmas from the infected DF-1 cell cultures, we determined the total cell-associated mycoplasmas as the percentage ratio of the colony forming units (CFUs) recovered from the cell fraction to the CFU present in the initial inoculum (Fig. S1A). Our results indicated differences in total cell-associated mycoplasmas between strains (one-way ANOVA: F_2,6_ = 109.8, p < 0.0001). Indeed, we found that 11.4% (±SE 0.4) of the initial inoculum of HF_1994 was cell-associated, which is higher than the 3.3% (±SE 0.05) for R_low (Tukey HSD test: p < 0.0001), and the 1.1% (±SE 0.2) for R_high (Tukey HSD test: p < 0.0001). There was, however, no significant difference between the percentages of cell-associated bacteria of R_low and R_high (Tukey HSD tests: p = 0.12). We found similar patterns when estimating levels of adhesion by subtracting the percentage of invaded mycoplasmas from the percentage of cell-associated mycoplasmas (one-way ANOVA: F_2,6_ = 15.5, p = 0.004; Fig. S1B). Indeed, HF_1994 then also adhered at higher levels than both R_low (mean ± SE: HF_1994 = 2.9 ± 0.4%, R_low = 0.9 ± 0.3%; Tukey HSD test: p = <0.01) and R_high (mean ± SE = 0.8 ± 0.1%; Tukey HSD test: p < 0.01), although there remained no difference between R_low and R_high (Tukey HSD test: p = 0.98). These results were consistent with our DIF results above and indicated a greater ability of HF_1994 to adhere to model avian cells than both R_low and R_high. Further complementary assays (for e.g., using direct labelling of *M. gallisepticum* strains) are, however, required to accurately quantify cell adherence and yield comprehensive insights into differences between the strains.

Finally, having established the ability of HF_1994 to invade, we conducted a time course gentamicin invasion assay to determine how rapidly HF_1994 was able to enter DF-1 cells (Fig. 2B). To do so, invasion of DF-1 monolayers was measured at 5, 15 and 60 min post-infection. We found that R_low and HF_1994 invaded DF-1 cells rapidly, with intracellular bacteria detectable as early as 5 min post-inoculation. R_high, on the other hand, was not detected at this early time point.

### HF_1994 shows high levels of persistence and exit from avian cells

In order to determine the post-infection fate of HF_1994 (i.e., whether it is capable of persistence and replication within the avian cell, and/or of exiting post invasion), we employed a modified version of the gentamicin invasion assay (Fig. S2). In this adapted assay, parallel plates of DF-1 cells were infected with R_low, R_high and HF_1994 for 60 min, allowing sufficient time for adherence and invasion (as established above). Cell association, persistence and exit were then determined in infected DF-1 cell cultures by subsequently treating them with (+Gm) or without (−Gm) gentamicin followed by incubation in antibiotic free media for 24 h.

**Figure 3:**
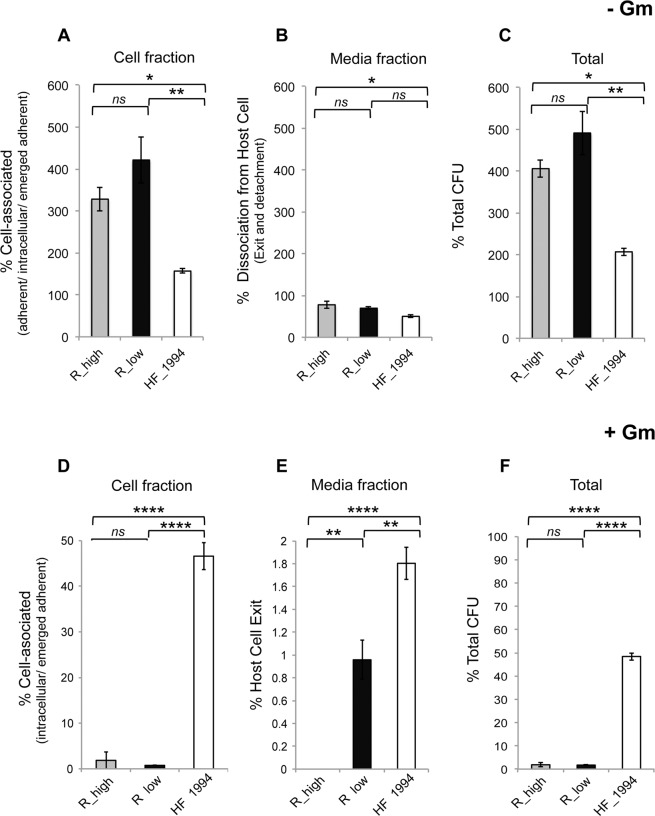
Post-infection fate of cell-associated and invaded *M. gallisepticum*. DF-1 monolayers were initially infected with R_high, R_low or HF_1994 for 60 mins. Extracellular, non-adherent bacteria were then eliminated by washing and incubation with **(A–C)** media minus gentamicin (−Gm) to investigate the post-infection fate of cell-associated *M. gallisepticum*, or with **(D–F)** gentamicin media (+Gm) to investigate the post-infection fate of invaded *M. gallisepticum*. Infected DF-1 were subsequently incubated in antibiotic-free media for 24 h to allow intracellular survival, replication and emergence. Percentage *M. gallisepticum* present in (**A**) the media fraction, and in **(B)** the cell fraction, relative to the average cell-associated mycoplasma present at 60 min post-infection. (**C**) Percentage relative total CFU from the combined fractions. Percentage of *M. gallisepticum* present in **(D**) the media fraction, and in **(E)** the cell fraction, at 24 h relative to the average number of intracellular *M. gallisepticum* present 60 min post-infection (point of gentamicin addition). (**F**) Total percentage CFU present at 24 h relative to the total CFU present post 60 min infection. Data represent mean ± SE. *Indicates p < 0.05, ** indicates p < 0.01, ***indicates p < 0.001, ***indicates p < 0.0001.

First, we tested the ability of *M. gallisepticum* bacteria to persist within and/or adhere to DF-1 cells by using the cell fraction harvested from the −Gm treatment. Indeed, this cell fraction contained mycoplasmas that have remained attached to the avian cell surface throughout the entire duration of the experiment (adherent), and/or that first invaded the intra-cellular space but then emerged from within the cell and adhered to the cell surface (emerged adherent) and/or that remain intracellular. We found a significant difference in the ability of the strains to persist within and/or adhere to DF-1 cells (one-way ANOVA: F_2,6_ = 13.7, p = 0.006), with HF_1994 being less able to do so than R_low (mean ± SE: HF_1994 = 156 ± 29%; R_low = 421.5 ± 56%; Tukey HSD test: p < 0.01) and R_high (mean ± SE = 328.2 ± 28.7%; Tukey HSD test: p < 0.05) (Fig. 3A). This suggests that the poultry strains were able to increase in association with the avian cell at higher levels than the house finch outbreak strain.

Second, we measured the ability of intra-cellular and adhered *M. gallisepticum* bacteria to be released in the extra-cellular environment by using the media fraction harvested from the −Gm treatment. Indeed, this media fraction contained *M. gallisepticum* bacteria that dissociated from DF-1 cells either after detaching from the surface of these cells, or after exiting the intracellular space (Fig. 3B). We found a significant difference between the strains in the overall number of bacteria released into the extra-cellular environment either after invading or remaining adhered to the surface of DF-1 cells (one way ANOVA: F = 6.4, p = 0.03). Indeed, HF_1994 exhibited a lesser number of bacteria released relative to R_high (mean ± SE: HF_1994 = 50 ± 4%, R_high = 78 ± 8%; Tukey HSD test p < 0.05), but not significantly less than R_low (mean ± SE: = 70 ± 4%; Tukey HSD test p = 0.11).

Third, we measured the replicative ability of intra-cellular and adhered *M. gallisepticum* in infected DF-1 cell cultures by comparing the total number of colonies in the combined cell and media fractions before (i.e., at 60 min post-infection) and after –Gm treatment (Fig. 3C). We found that all strains were able to replicate, as evidenced by increases in the number of CFU present at 24 h of the –Gm treatment compared to that present at 60 min. However, there was a significant strain effect on the ability to replicate (one-way ANOVA: F_2,6_ = 19.7, p = 0.002). Indeed, replication was highest for R_low (mean ± SE = 491.1 ± 52.0%), intermediate for R_high (mean ± SE = 405.9 ± 21.1%) and lowest for HF_1994 (mean ± SE = 206.9 ± 9.4%), although the total number of CFU isolated still indicates that the population had, at minimum, doubled over the course of the experiment. Between-group comparisons revealed no significant difference between R_high and R_low (Tukey HSD test: p = 0.24, but significant differences between R_high (= 405.9 ± 21.1%) and HF_1994 (Tukey HSD test: p < 0.05), and between R_low and HF_1994 (Tukey HSD test: p < 0.01).

Fourth, we measured the ability of *M. gallisepticum* to persist and replicate within infected avian cells only by using the cell fraction harvested from the +Gm treatment, since it should contain: (i) the intracellular (i.e., non-exited) *M. gallisepticum*, and (ii) the *M. gallisepticum* that emerged from the avian cells and remained attached to the avian cell membranes (i.e., emerged adherent) during incubation in antibiotic-free media. We found a significant difference in within-cell persistence over time between strains (one-way ANOVA: F_2,6_ = 421.7, *p* < 0.0001), with almost half of the HF_1994 present at 60 min post-infection persisting for a further 24 h (mean ± SE = 45.8 ± 1.9%), which was significantly higher than found with R_low (mean ± SE = 0.8 ± 0.01%; Tukey HSD test p < 0.0001) (Fig. 3D).

Finally, we measured the bacteria’s ability to exit avian cells using the media fraction harvested from the +Gm treatment, since it should only contain *M. gallisepticum* released from the intracellular space during incubation in antibiotic-free media. We found a significant difference in the proportion of exited intracellular bacteria between strains (one-way ANOVA: F_2,6_ = 77.14, p < 0.0001). Indeed, a significantly higher proportion of intracellular HF_1994 were found to have exited avian cells in a viable state relative to R_low (mean ± SE: HF_1994 = 1.9 ± 0.2%, R_low = 0.9 ± 0.2%; Tukey HSD test: p < 0.01). In other words, HF_1994 is not only capable of avian cell exit, but that it can also do so to a greater extent than R_low (Fig. 3E). No viable CFU were recovered for R_high, indicating that the small number of mycoplasmas of this strain that had successfully invaded DF-1 cells lacked the capability to exit these avian cells. However, the total CFU of HF_1994 and R_low bacteria measured following gentamicin treatment were lower than the number measured before treatment, suggesting that neither bacterial strain was replicating within the intracellular environment (Fig. 3F).

### HF_1994 is not cytotoxic towards avian cells

**Figure 4:**
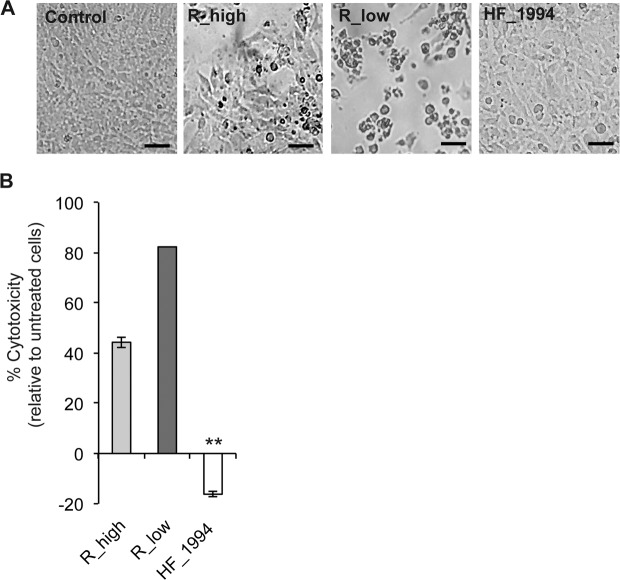
Cytotoxicity of *M. gallisepticum* strains towards DF-1. Monolayers of DF-1 cells were inoculated with R_low, R_high or HF_1994 and incubated for 24 h. (**A**) Light microscope image panels of cell monolayers following 24 h infection, from left to right: control (untreated) cells, cells infected with R_high, R_low and HF_1994 (Scale bars = 20 μm). (**B**) % Cytotoxicity in DF-1 infected with HF_1994 is significantly lower than in untreated control cells (*p* < 0.001). ** indicates *p* < 0.001.

Differences in cytotoxicity between the house finch and poultry strains of *M. gallisepticum* were first determined using light microscopy by examining the morphology of infected DF-1 cell monolayers 24 h after infection and comparing them to those of control cell monolayers (Fig. 4A). While control cells were present as a confluent monolayer, high levels of cytotoxicity could be observed in cells infected with R_low, as evidenced by the fact that the majority of infected DF-1 cells infected showed marked cell shrinkage, rounding and loss of adherence. In contrast, DF-1 cells infected with either R_high or HF_1994 showed little evidence of cytotoxicity, with monolayers remaining largely intact in both cases. DF-1 cells infected with HF_1994 actually appeared enlarged relative to both control and R_high-infected cells, suggesting not only that HF_1994 was not cytotoxic, but that it even improved the viability of DF-1 cells.

The viability of infected DF-1 at 24 h was further assessed using the lactate dehydrogenase (LDH) assay (Fig. 4B). The level of cytotoxicity was> 80% for R_low-infected cells and ~40% for R_high-infected cells. As above, we found that HF_1994 was not cytotoxic towards DF-1 and, in fact, even appeared to reduce the percentage cytotoxicity relative to control untreated cells (one-way ANOVA: F_3,20_ = 1496, p < 0.0001). This supports a role of HF_1994 in prolonging survival of the infected confluent cell monolayer from the background levels of natural cell death occurring over time in the untreated control cells.

## Discussion

Our results show that the bacterial strain sampled at epizootic outbreak in the novel house finch host (HF_1994) was able to adhere to and quickly invade cultured, non-phagocytic chicken embryonic fibroblasts (DF-1) cells. This ability to invade DF-1 cells was greater than that of the model poultry R-type strain (R_low) and the attenuated derivative (R_high). In support for high levels of invasion at epizootic outbreak in house finches, two further early strains (HF_1995 and HF_1996) were found to display similarly high invasion abilities of DF-1 cells. In addition, when considering only the bacteria that had invaded DF-1 cells, we found that HF_1994 displayed greater levels of intracellular persistence over time, as well as a greater ability to exit DF-1 cells relative to the virulent poultry strain, R_low. Despite this, neither HF_1994 nor R_low evidenced an increase in colony counts over time, suggesting that they were not replicating within the intra-cellular environment. Finally, we found that HF_1994 not only displayed lower levels of cytotoxicity towards DF-1 cells than the poultry strains, but H_1994 actually improved DF-1 cell survival relative to non-infected control DF-1 cell monolayers. Taken together, our results provide a novel picture of a bacterial pathogen at epizootic outbreak and suggest that, following its jump into the novel house finch host, *M. gallisepticum* may have been particularly adept at exploiting the host intra-cellular space. Whether the high avian cell invasion phenotype of the outbreak strain in chicken embryonic fibroblasts is also displayed in interactions with house finch cells, and whether such phenotype  played a role in the host shift, remains to be investigated.

While the adherence of bacterial pathogens to host cells and tissues is necessary for the initiation of infection and colonization of the host (reviewed in^47–50^), invading the intracellular environment by becoming internalized into non-phagocytic host cells can enable bacterial pathogens to pass through epithelial barriers and escape immunity^47^. In *M. gallisepticum*, cytoadhesion is thought to occur via a specialized organelle, termed tip organelle or terminal structure, which is localized at the tip of their elongated flask-cell shape and packed with adhesion molecules that bind the eukaryotic cell membrane^51^. Several adhesins have been identified in *M. gallisepticum* and notably, genes encoding the cytadhesins GapA, CrmA, CrmB, MGA_0205, Hlp3, PlpA and Lpd were shown to have diverged in the epizootic outbreak strain relative to the poultry strain R_low^31^. GapA and CrmA have also been linked to *M. gallisepticum*’s ability to invade both avian and non-avian host cells^52–54^. Given our finding that HF_1994 is qualitatively more adhesive and quantitatively more invasive of DF-1 cells than R_low, it is tempting to speculate that these genetic changes at key cytadhesins and putative invasins in the house finch epizootic outbreak strain underlie differences in the ability to adhere and invade DF-1 cells. Further work is required to determine whether this is, indeed, the case. Regardless, these results also give rise to the hypothesis that a high cell adhesion, high cell invasion phenotype may be beneficial at colonisation of a novel, hostile host environment.

Little is known about where mycoplasma bacteria reside and whether they are able to replicate in the intracellular environment^49^, although vertebrate cell invasion has been shown to induce systemic changes at the genomic, proteomic and metabolic levels in *M. gallisepticum*^44^. Nearly all invasive bacteria enter a membrane bound vacuole^47^ and intracellular *M. penetrans* has accordingly been localized within endosomes, where it is thought to combat lysosomal oxidative stress through the production of antioxidants^55^. The lack of biosynthetic pathways in mycoplasma means that they are dependent on their host for the supply of resources necessary for replication^56^. However, while R_low has been shown to upregulate the expression of genes associated with translation upon exposure to vertebrate cells^57^, ours and a previous study both show that the number of internalized R_low bacteria decreases 24 h post infection^22^, suggesting that this pathogen may not be replicating intracellularly. We found that intracellular HF_1994 similarly decreased in numbers over 24 h, but to a much lesser extent than R_low, suggesting that HF_1994 is equally unlikely to replicate intracellularly, but that it can persist within the avian cell for longer periods of time than R_low. Regardless of whether or not HF_1994 is able to replicate within avian cells, our results suggest that the virulence of HF_1994 is unlikely to derive from high replication rates and may instead stem from high levels of cell invasiveness and cell exit.

Intracellular pathogens can exit host cells in a variety of ways, although the precise mechanisms remain largely unknown^58^. For example, they can induce lysis and extrusion (e.g *Chlamydia trachomatis*^59^) or apoptosis (e.g. *Francisella tularensis*^60^), hijack the host cell machinery (e.g., *Shigella flexneri*^61^), or spread into a neighboring cell through protrusion into that cell and subsequent engulfment (e.g *Listeria monocytogenes*^62^). Host cell membrane protrusions containing *M. hominis* have indeed been observed in infections of HeLa cells, suggesting exit via exocytosis^63^. Pore-forming proteins are also frequently seen to be involved in escape from the vacuole, prior to replication within the cytoplasm and subsequent cell exit^58,64^. *M. gallisepticum* R_low^22^ has previously been shown capable of persistence within, and exit from, host cells and a motif similar to that found in the pore-forming aerolysin toxin has been found in four *M. gallisepticum* proteins, including GapA^35^. While the precise mechanisms of host cell exit in *M. gallisepticum* remain to be determined, our study reveals that HF_1994 was more successful in exiting avian cells than R_low, suggesting that intracellular stages of infection benefited infection establishment and persistence in the novel finch host.

Bacterial pathogens will, in some instances, induce host cell death to exit the intracellular environment^58^, but in other cases, they will instead prevent programmed host cell death to favour intracellular persistence and replication^65^. Such latter anti-apoptotic effect is, for example, observed in chronic infections of *Mycobacteria tuberculosis*^65^, and in enteropathogenic *Escherichia coli*, where the expression of the EspZ protein delays cell death in infected rabbit epithelia and is essential for virulence^66,67^. *Staphylococcus aureus* has also been shown to exert a cytoprotective effect on invaded host cells, possibly through the upregulation of anti-apoptotic genes^68^. Many mycoplasmas are known to live in close association with host cells without causing cytotoxic effects; for example, cell culture infections can go unnoticed for long periods, and human and animal infections are often chronic and persistent^49^. Such infections, as with *M. arginini*^69^, *M. fermentans and M. penetrans*^70^, have been associated with the activation of nuclear factor (NF)-kB, and suppression of p53 and p21, resulting in the inhibition of apoptotic cell death. On the other hand, mycoplasmas can sometimes be extremely cytotoxic to their hosts; for instance, our study confirms previous findings that R_low causes high levels of mortality in DF-1 cells at 24 h and 100% mortality 48 h post-infection^22^. This cytotoxicity by R_low has been associated with the lipoproteins MslA and MGA_0676 *in vivo*^20,71^. Interesting, mycoplasmal lipoproteins can also be associated with anti-cell death phenotypes^72^ and we know that extensive genomic differences have been identified in the phase-variable lipoproteins (VlhA) between the sequenced isolate of the outbreak strain and R_low^31^. The cytotoxicity of some mycoplasma species has also been linked to their production of hydrogen peroxide (H_2_O_2_). For instance, *Mycoplasma mycoides-*induced cytotoxicity is dependant on high levels of H_2_O_2_ production and adhesion to host cells that facilitates translocation of H_2_O_2_ into the cytoplasm^73^. Similarly, elevated production of H_2_O_2_ occurs during intracellular infection by *M. gallisepticum* S6^44^, while H_2_O_2_ production by R_low causes cytotoxicity to eukaryotic cells in culture, but is not required for pathogenicity *in vivo*^38^. Our results reveal that HF_1994 exerted measurable effects on prolonging the survival of DF-1 cells, but whether this effect is the result of the expression of different or of altered lipoproteins, or indeed is related to decreased H_2_O_2_ production, remains to be determined.

Our study of a bacterial pathogen isolated at epizootic outbreak in a novel host has enabled us to characterise the ability of an emerging avian pathogen to interact with model avian cells. Specifically, we found that the outbreak strain (HF_1994) has an increased ability to invade and exit from non-phagocytic avian cells relative to a reference virulent poultry strain (R_low), suggesting that, at emergence, this pathogen was highly adept at using the host intracellular space. The virulence of *M. gallisepticum* has been shown to have increased subsequently over the course of the epizootic in house finches^40,41^. Whether virulence evolution was associated with changes in host cell invasion ability, particularly as resistance spread within the house finch host population^42^, remains to be determined and represents an exciting focus for future research.

## Materials and Methods

### Mycoplasma culture and infection

The *M. gallisepticum* poultry strains R_low (virulent) and R_high (attenuated) used in this study were kindly provided by Dr Michael Szostak (Institute of Microbiology, Department of Pathobiology, University of Veterinary Medicine, Vienna). A low passage isolate (cryopreserved at two passages post isolation, as soon as enough culture had grown to make a freezer stock) of HF_1994 (also known as S11 or VA_94) was used in all assays (collected in Virginia in June 1994 and archived in Professor Hill’s laboratory). Poultry and House finch strains of *Mycoplasma gallisepticum* were cultured in SP4 media supplemented with 0.1% phenol red (Sigma) as a growth indicator. Cultures were grown to mid-exponential phase as determined by colour shift of the media from pink/red to orange. In order to prepare inoculum for the assays, the cultures were pelleted by centrifugation at 10,000 × *g* for 10 minutes, resuspended in DMEM and passed through a sterile 23-gauge needle approximately 5 times in order to disperse mycoplasmal cell aggregates without compromising viability. The prepared *M. gallisepticum* isolate suspensions were then inoculated into the required assay. The number of viable mycoplasmas used in the infection experiments was determined by plating serial dilutions of the inoculum on SP4 1% agar plates. *M. gallisepticum* inoculum was prepared for all experiments following the same method.

### Cell culture

The Chicken Embryonic Fibroblast cell line UMNSAH/DF-1 (ATCC^®^ CRL-12203™) was obtained from the European Cell and Culture Collection (ECACC). The DF-1 cells were maintained in Dulbecco’s Modified Eagle’s Medium (Cat # 31966, Gibco) supplemented with 10% heat-inactivated foetal bovine serum at 38 °C, 5% CO_2_. Cell morphology was assessed and imaged through an inverted light microscope (Leica DM IL LED and LAS X software). DF-1 cell cultures were routinely screened for mycoplasma contamination by directly plating onto SP4 agar plates and monitoring for colony formation and also by specific DNA staining with DAPI and analysis via fluorescence microscopy.

### Adherence and invasion assays

The differential immunofluorescence (DIF) assay was used to qualitatively identify and visualize intracellular *M. gallisepticum*. The DIF assay procedure was carried out as described previously^43,45^. In brief, confluent monolayers of DF-1 cells grown on sterile glass coverslips were inoculated with *M. gallisepticum* strains at a multiplicity of infection of ~20 (as determined by plating a dilution series of the inoculum onto SP4 agar plates in order to determine viable cfu per ml inoculated onto the DF-1 monolayers) and incubated at 38 °C/5% CO_2_ for 16 h. Cells were washed in 2% bovine serum albumin (BSA) in phosphate buffered saline solution (PBS) prior to incubation with the primary antibody: 1 in 300 polyclonal rabbit anti-*M. gallisepticum* (orb10563, Biorbyt, Ltd) in 2% BSA/PBS for 30 minutes at room temperature. Cells were then washed and incubated with secondary antibody FITC-conjugated goat anti-rabbit (Invitrogen) for 30 min to stain extracellular mycoplasmas. Coverslips were washed 3 times in 1X PBS in order to remove antibodies prior to methanol permeabilisation (1 min at −20 °C). Cells were then washed as previously and incubated again with primary anti-*M. gallisepticum* as previously and washed before incubation with Alexa-Fluor 555 – conjugated goat anti-rabbit secondary antibody (1:500) to stain intracellular and extracellular mycoplasmas. Finally, cells were washed and then mounted with ProLong Diamond Antifade reagent plus DAPI (Molecular Probes). Coverslips were observed (Leica TCS confocal system) inverted confocal microscope. Extracellular and intracellular mycoplasmas were detected using FITC and Y3 filter sets and imaged. Images were merged in order to determine cellular location of mycoplasma, extracellular mycoplasmas appear yellow (overlay of red and green) and intracellular mycoplasmas appear red.

The gentamicin invasion assay was carried out as described previously^43,46^. Confluent monolayers of DF-1 cells were grown in 24 well plates prior to inoculation with *M. gallisepticum* strains for time points ranging from 5 minutes to 60 min as required at 38 °C, 5% CO_2_. Following the infection period the DF-1 monolayers were washed with 1X PBS in order to remove non-adherent mycoplasmas prior to treatment with gentamicin media (400 μg ml-1 gentamicin in DMEM) for 3 h at 38 °C, 5% CO_2_. The gentamicin media was then removed and the DF-1 monolayers washed 3 X with PBS before trypsinisation in order to harvest the cells. Harvested DF-1 cells were resuspended in SP4 media, and serial dilutions plated directly onto SP4 agar plates in order to establish the number of CFU. The infected avian cell suspensions were plated directly since mycoplasmas are as sensitive to the detergent lysis typically used in such assays as the avian cells themselves^74^. Plates were incubated at 37 °C for 7 to 10 days and mycoplasma colonies were then counted. Untreated DF-1 cells were included as a control, additionally, for confirmation of killing by the gentamicin solution aliquots of mycoplasma inocula were treated with this solution under the same conditions as the invasion assay with and plated out. No viable CFU were detected in these controls indicating that the gentamicin solution was effective, and that there was no background mycoplasmal contamination of the DF-1 cells. Data is representative of three independent experiments performed in duplicate.

### Persistence and avian cell exit assays

Modified gentamicin assays were conducted in order to assess the ability of *M. gallisepticum* strains to persist within and/or exit the avian cell following invasion, as outlined in the schematic workflow diagram (Fig. S2). DF-1 monolayers, seeded into parallel 24 well plates, were inoculated with *M. gallisepticum* strains R_high, R_low and HF_1994 (Fig. 3A). The *M. gallisepticum* strains were then incubated with the DF-1 cells for 60 mins in order to establish cell infection, as described above (Fig. 3B). Subsequently, the infected DF-1 cells were washed with sterile 1X PBS to remove non-adherent mycoplasmas, and incubated for 3 h either with gentamicin media (+Gm) to kill adherent mycoplasmas, or antibiotic-free media (−Gm), retaining all cell-associated bacteria, both invaded and adhered (Fig. 3C). Following the gentamicin +/− treatment step the media was removed and the monolayers washed with 1X PBS. At this point a control set of replicate wells was harvested and plated in order to establish the total invaded bacteria in the +Gm treatment, and total cell-associated bacteria (adhered and invaded) in the −Gm treatment at 60 mins. The remaining parallel assay plates of infected DF-1 cells were overlaid with antibiotic-free DMEM and incubated for 24 h at 38 °C/5% CO_2_. Following the 24 h incubation step, media and cell fractions from the parallel infection plates were harvested (Fig. 4D). Media from +Gm (invaded only) treated wells was harvested and plated out, as described above, in order to determine avian cell exit of invaded mycoplasmas. Cells from +Gm treatment were trypsinised, harvested and plated out to determine the presence of intracellular bacteria at 24 h. Media from –Gm treated cells (adhered and invaded) was harvested and plated out to determine total viable *M. gallisepticum* released from adherent and intracellular mycoplasmas. Cells from −Gm treated cells were plated out to determine the total number cell associated mycoplasmas present (adhered and invaded).

### Cytotoxicity assay

The cytotoxicity of *M. gallisepticum* strains towards DF-1 cells was assessed using the CytoTox 96^®^ Non-Radioactive Cytotoxicity Assay Kit (Cat #G1780, Promega). DF-1 cells were seeded at 10^5^ in V-bottomed 96 well culture plates (Corning). Wells were prepared in triplicate for experimental samples: DF-1 infected with R_low, R_high and HF_1994, at a multiplicity of infection (MOI) of approximately 20, and for control samples: untreated cells (for background/spontaneous LDH release), untreated cells lysed (total maximum LDH), culture medium (for culture medium background control) and culture medium plus *M. gallisepticum* (for bacterial LDH background control). DF-1 were infected *M. gallisepticum* strains and incubated for 24 h. Forty-five minutes ahead of cell harvest Lysis Solution (10×) was added to the total maximum LDH release control cells. The assay plate was then centrifuged at 250 g for 4 minutes in order to pellet cells and free mycoplasmas. Fifty microliters of the resulting supernatant was then transferred to the corresponding well of a flat bottom 96 well plate (taking care not to disturb the cell pellet) and 50 μl of reconstituted substrate mix (as manufacturer’s instructions) added. Plates were incubated in the dark for 20 minutes and the reaction stopped using 50 μl of the Stop Solution provided with the kit. Absorbance readings were made at 490 nm (Molecular Devices SpectraMax). Background was subtracted from the experimental samples using the control medium containing *M. gallisepticum* sample values. Percentage cytotoxicity was calculated as follows: % cytotoxicity = (Infected cells − Untreated cells)/(Total Maximum LDH − Untreated cells) × 100.

### Statistical analysis

All statistical analyses were conducted in R^75^. We tested for differences in the ability of the *M. gallisepticum* strains HF_1994, R_low and R_high to: associate with, invade, adhere to and exit DF-1 cells by running one way ANOVA with percentage cell association, invasion, adherence or avian cell exit (as appropriate) as the response variable and *M. gallisepticum* isolate (i.e. HF_1994, R_low or R_high) as the explanatory term. *Post hoc* comparisons were carried out using the Tukey HSD test. Differences in cytotoxicity were modelled using a one-way ANOVA, with percent cytotoxicity (relative to untreated cells) as the response variable, and with cell treatment (i.e. infection with HF_1994, R_low, R_high or untreated control) as the explanatory term. All figures were made using ggplot2^76^.

## Supplementary information

Supplementary information.
