# Supplementary material for: Multiple differences in pathogen-host cell interactions following a bacterial host shift
Source: Sci Rep. 2020 Apr 22;10:6779. doi: 10.1038/s41598-020-63714-0 (PMC7176683; doi:10.1038/s41598-020-63714-0)

**Supplementary Information**

Multiple differences in pathogen-host cell interactions following a bacterial host shift

A. J. Dowling, G. E. Hill and C. Bonneaud

**Fig S1.** ***M. gallisepticum* HF_1994 is highly adherent of non-phagocytic avian cells relative to poultry strains. (A)** Percent CEF-associated mycoplasmas relative to the initial inoculum following 1h infection. Significantly higher levels of HF_1994 are associated (i.e., adhered and invaded) with avian cells (CEF) in comparison to R_low (p < 0.01). (B) Percentage adherence of mycoplasmas indicated by subtraction of % invaded mycoplasma from % cell associated mycoplasma following 1h infection.


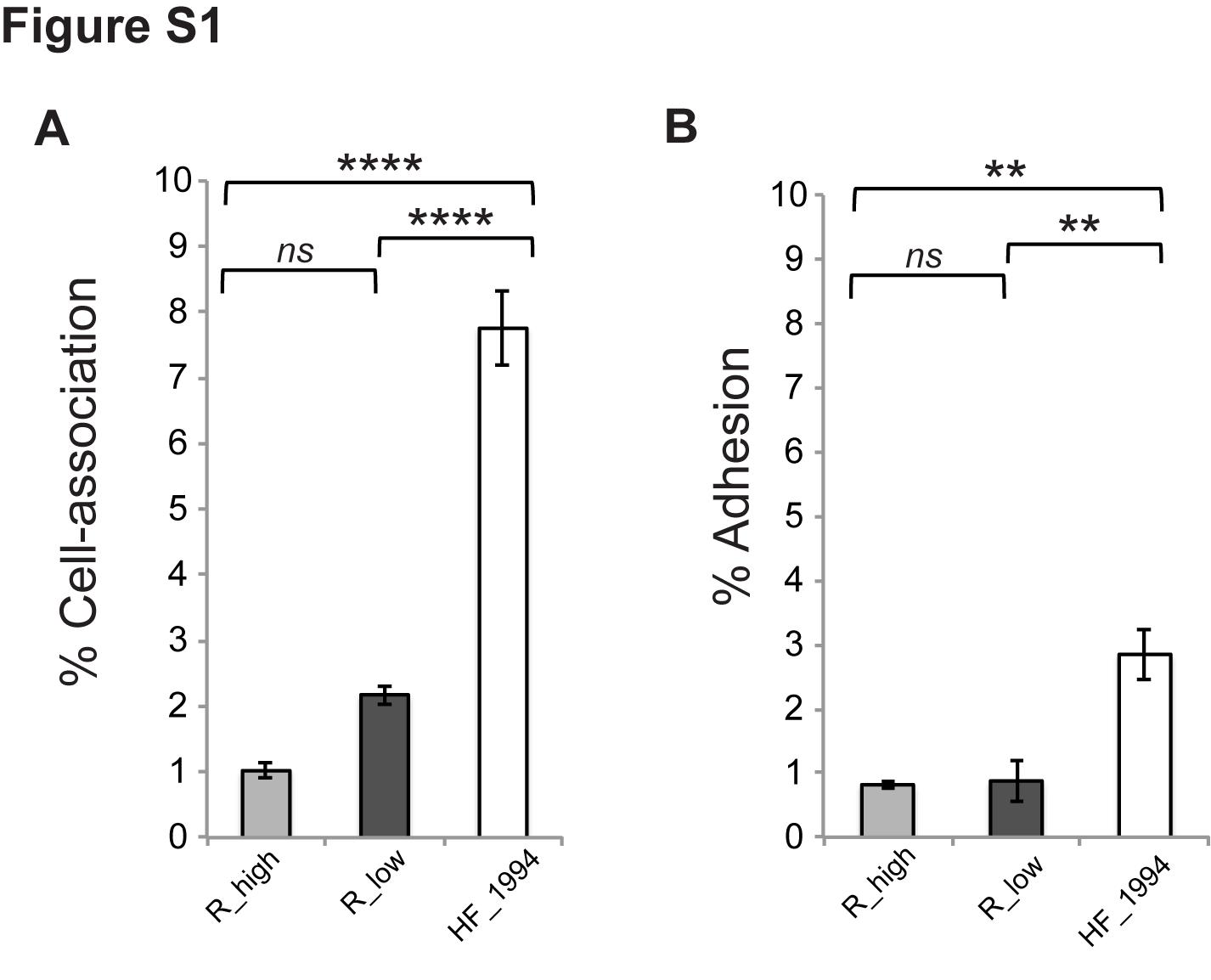


**Fig S2. Workflow diagram of post infection fate assay.** Schematic representation of the modified parallel gentamicin protection assay workflow used to investigate intracellular survival and avian cell exit of *M. gallisepticum* strains. (**A)**. CEF monolayers seeded into parallel 24 well plates are inoculated with *M. gallisepticum* strains R_high, R_low or HF_1994.The *M. gallisepticum* strains are incubated with the CEF cells for 60 mins in order to establish cell infection. Green dots indicate free mycoplasmas in the extracellular media, Blue dots indicate mycoplasmas adhered to the avian cells, and Red dots illustrate bacteria that have invaded. (**B)**. CEF monolayers are washed to remove non-adherent mycoplasmas. (**C).** Parallel wells infected with the same isolate are subjected to two different treatments, one set is treated with media containing gentamicin (+ Gm) in order to kill extracellular adherent bacteria, and the second set with media containing no gentamicin for 3 h (- Gm). Following the gentamicin +/- treatment step the media is removed, the monolayers are washed and a sub set of replicate wells harvested and plated in order to establish total invaded bacteria in the + Gm treatment, and total cell-associated bacteria (adhered and invaded) in the – Gm treatment. In the experimental assay plate fresh antibiotic-free media is added to the wells and the cells are then incubated for 24 h. (**D).** Harvesting and plating – media from +Gm (invaded only) treated wells is harvested and plated out to determine avian cell exit of invaded mycoplasma (free red circles). CEF cells from +Gm treatment are trypsinised harvested and plated out to determine the presence of intracellular (red dots), or emerged adherent bacteria (attached red circles) at 24h. Media from –Gm treated cells (adhered and invaded) is harvested and plated out to determine total viable *M. gallisepticum* released from both those adhered to (blue circles) and invaded (red circles). –Gm treated CEF cells are plated out to determine the total number of cell-associated mycoplasmas present (adhered and invaded) at 24h, also including *M. gallisepticum* which may emerge from the avian cell but remained attached.


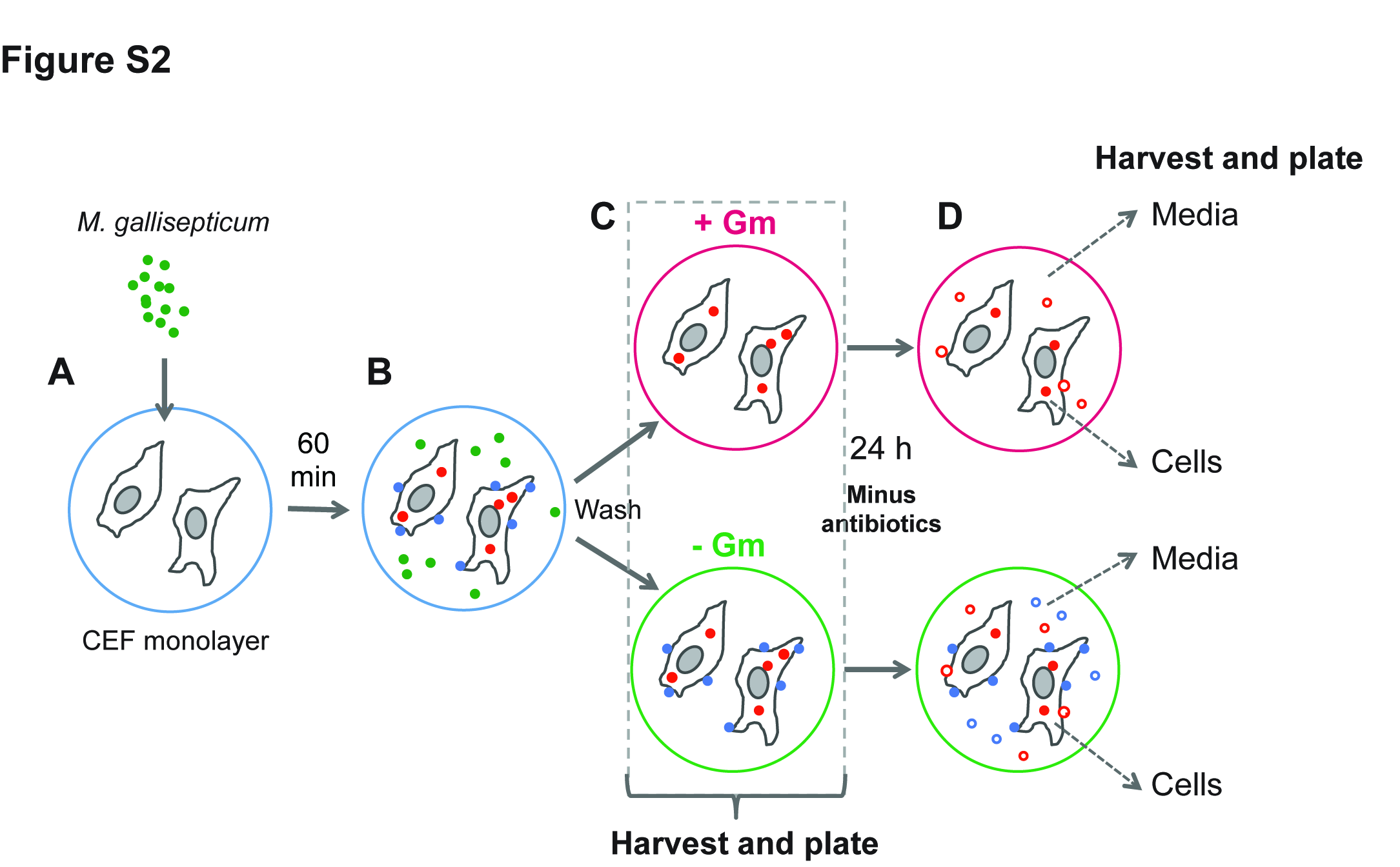

Supplement: Supplementary file 1 — Supplementary information. [file 41598_2020_63714_MOESM1_ESM.docx]
